# Supplementary material for: Longitudinal fragility phenotyping contributes to the prediction of lifespan and age-associated morbidity in C57BL/6 and Diversity Outbred mice
Source: GeroScience. 2024 Jun 27;46(5):4937–54. doi: 10.1007/s11357-024-01226-9 (PMC11639350; doi:10.1007/s11357-024-01226-9)
Supplement: Supplementary file 1 — (pdf 2079 KB) [file 11357_2024_1226_MOESM1_ESM.pdf]

Supplemental Materials for 'Longitudinal Fragility Phenotyping Contributes to the Prediction of Lifespan and Age-Associated Morbidity in C57BL/6 and Diversity Outbred Mice'

Alison Luciano[1], Laura Robinson[1], Gaven Garland[1], Bonnie Lyons[1], Ron Korstanje[1], Andrea Di Francesco[2], Gary A. Churchill [1,*]

[1]The Jackson Laboratory, Bar Harbor, ME, USA

[2]Calico Life Sciences LLC, South San Francisco, CA, USA

[*]Correspondence: gary.churchill@jax.org

eFigure 1: Locally weighted smoothed mean frailty by index item. X-axis: PLL range observed, 0.6 to 1.0. Y-axis: Locally weighted smoothed mean frailty. The 20 individual frailty items highlighted showed weak correlation with life expectancy in the DO cohort; remaining items and color legend displayed in Figure 2.

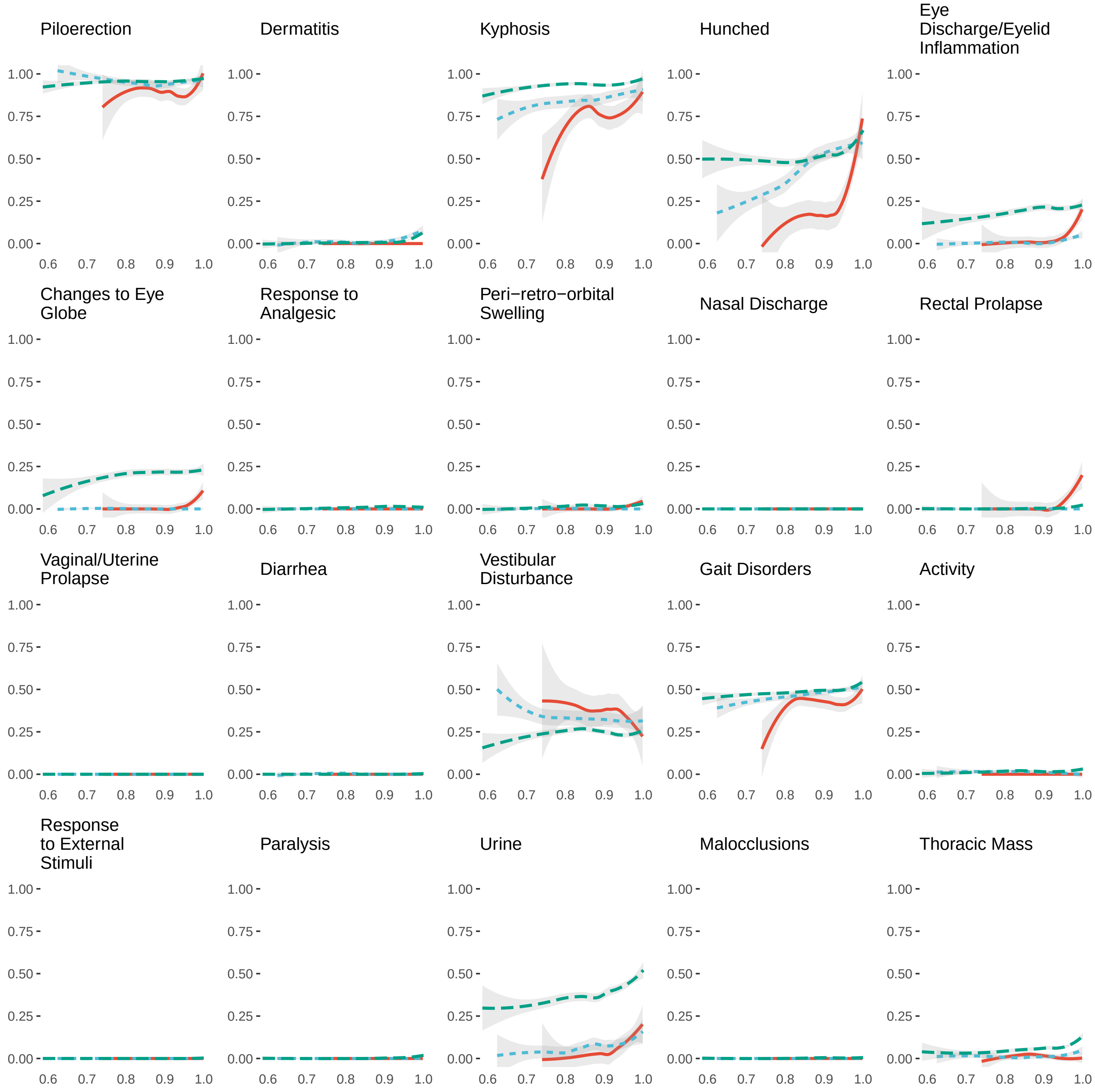

eFigure 2: Feature importance. A) Stacking coefficients are used to weight the predictions from each model in the ensemble. A parsimonious model with only age and dietary assignment was weighted highly. B) Variable importance*. Variables listed near the top of the figure are most important for 95PLL prediction in this model. C) Dependence plot*. Machine learning algorithm identified monotonic nonlinear association between age in weeks and 95PLL without pre-specifying non-linearity. D) Variable importance** E) Dependence plot**; Identified nonlinear relationship with frailty score where scores 2SD below mean are more informative than scores ˜-1.5SD below mean, and increasing scores are positively associated with importance thereafter. High frailty score (>=1.5SD) from mean is most informative in 95PLL prediction). F) SHAP interaction values for age and recent frailty change**. Visualizations generated with R package SHAPforxgboost Liu *et al.* [1]. *determined via SHAP values computed for XGB algorithm with the highest ensemble weight (rank 1; eFig 2A) **determined via SHAP values computed for XGB algorithm with a frailty feature with the highest ensemble weight rank (rank 3; eFigure 2A)

A

Rank	Model	PP	Weight
1	XGB	m0	5.400
2	RF	m3	1.431
3	SVM	m1	0.915
4	XGB	m3	0.732
5	SVM	m3	0.729
6	SVM	m1	0.340
7	RF	m0	0.310
8	SVM	m1	0.261
9	SVM	m3	0.189
10	SVM	m0	0.114

B

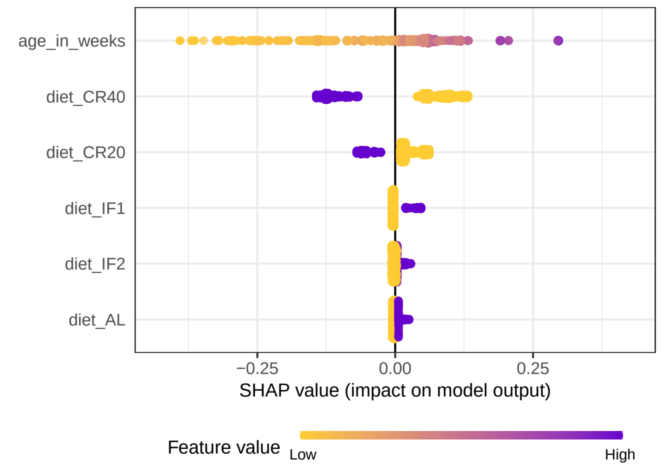

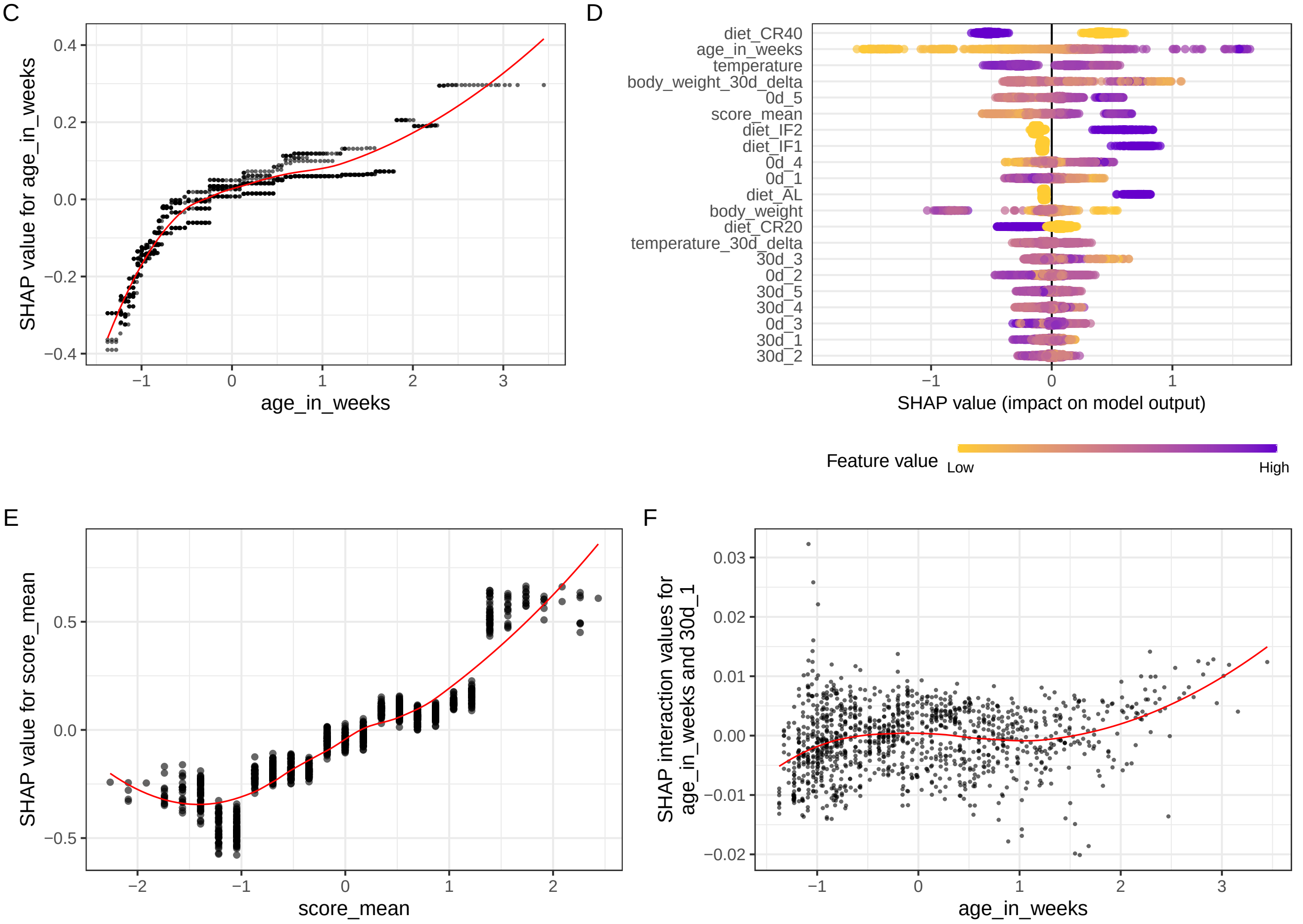

eTable 1: Fragility items.

System and Item	Potential Deficits
**Digestive**	
Diarrhea	Soft feces
Malocclusions	Uneven and/or overgrown exposed teeth
Rectal Prolapse	Rectal tissue exposed
**Discomfort**	
Piloerection	Widespread piloerection
Head Piloerection[a]	Piloerection across the head
Response To Ocular Analgesic	Squinting unresolved
**Integument**	
Coat Condition	Coat is unkempt
Dermatitis	Skin lesions
Pallor and/or Cyanosis	Pale or cyanosis of skin
Dehydration/Skin Turgor	Tented skin return to normal $>$2 seconds
**Physical/Musculoskeletal**	
Distended Abdomen	Bulding of abdomen caudal to ribcage
Tumors	Visible or palpable mass
Thoracic Mass[a]	Visible or palpable mass
Body Condition	Body Condition Score 3 or less
Tremor	Tremor at rest
Kyphosis	Dorsal curvature of the spine
Hunched	Dorsal curvature of the spine with abdominal tuck
Gait Disorders	Hopping, wobbling, circling, wide stance or weakness
Tail Stiffening	Tail unresponsive when stroked
Paralysis	Paralysis of $>$1 limb
**Ocular/Nasal**	
Nasal Discharge	Nasal discharge, both nares
Eye Discharge or Swelling	Eye bulging and/or secretions
Changes to the Globe	Clouding and/or spotting of cornea or enlarged globe
Peri Retro Orbital Swelling	Swelling around eye $>$5mm
**Urogenital**	
Urine	Bladder expression absent with massage
Vaginal/Uterine Prolapse	Vaginal/uterine tissue exposed
**Respiratory**	
Breathing Rate/Depth	Abnormal rate or effort, gasping
**Vestibulocochlear**	
Vestibular Disturbance	Head tilt, spinning, circling, head tuck, or trunk curling
**Behavior**	
Activity	No movement detected when placed in new cage
Response to External Stimuli	No response when lateral thorax nudged with a cotton tipped applicator

[a]added to the index based on summary observations after the first six of 200 collection dates; rectal body temperature and body weight also measured; systems categories correspond to those in Whitehead *et al.* [2]

eTable 2: Cumulative incidence of frailty indicators by diet in aged J:DO mice

	AL (N=25)	IF1 (N=33)	CR20 (N=55)	IF2 (N=48)	CR40 (N=73)	P-value
Activity						
Absent	14 (56.0%)	24 (72.7%)	28 (50.9%)	31 (64.6%)	41 (56.2%)	0.0345
Mild	7 (28.0%)	8 (24.2%)	24 (43.6%)	15 (31.3%)	32 (43.8%)	
Severe	4 (16.0%)	1 (3.0%)	3 (5.5%)	2 (4.2%)	0 (0%)	
Body Condition						
Absent	1 (4.0%)	0 (0%)	2 (3.6%)	2 (4.2%)	0 (0%)	<0.001
Mild	8 (32.0%)	13 (39.4%)	8 (14.5%)	9 (18.8%)	3 (4.1%)	
Severe	16 (64.0%)	20 (60.6%)	45 (81.8%)	37 (77.1%)	70 (95.9%)	
Breathing Rate/Depth						
Absent	1 (4.0%)	2 (6.1%)	1 (1.8%)	5 (10.4%)	2 (2.7%)	0.061
Mild	21 (84.0%)	24 (72.7%)	40 (72.7%)	41 (85.4%)	60 (82.2%)	
Severe	3 (12.0%)	7 (21.2%)	14 (25.5%)	2 (4.2%)	11 (15.1%)	
Changes to Eye Globe						
Absent	10 (40.0%)	19 (57.6%)	20 (36.4%)	24 (50.0%)	19 (26.0%)	0.0035
Mild	6 (24.0%)	7 (21.2%)	10 (18.2%)	5 (10.4%)	8 (11.0%)	
Severe	9 (36.0%)	7 (21.2%)	25 (45.5%)	19 (39.6%)	46 (63.0%)	
Coat Condition						
Absent	0 (0%)	0 (0%)	0 (0%)	0 (0%)	0 (0%)	0.012
Mild	3 (12.0%)	0 (0%)	4 (7.3%)	3 (6.3%)	0 (0%)	
Severe	22 (88.0%)	33 (100%)	51 (92.7%)	45 (93.8%)	73 (100%)	
Dehydration, Skin Turgor						
Absent	2 (8.0%)	7 (21.2%)	6 (10.9%)	8 (16.7%)	3 (4.1%)	0.058
Mild	0 (0%)	0 (0%)	0 (0%)	0 (0%)	0 (0%)	
Severe	23 (92.0%)	26 (78.8%)	49 (89.1%)	40 (83.3%)	70 (95.9%)	
Dermatitis						
Absent	20 (80.0%)	25 (75.8%)	47 (85.5%)	37 (77.1%)	57 (78.1%)	0.309
Mild	3 (12.0%)	7 (21.2%)	4 (7.3%)	8 (16.7%)	15 (20.5%)	
Severe	2 (8.0%)	1 (3.0%)	4 (7.3%)	3 (6.3%)	1 (1.4%)	
Diarrhea						
Absent	24 (96.0%)	32 (97.0%)	55 (100%)	47 (97.9%)	73 (100%)	0.161
Severe	1 (4.0%)	1 (3.0%)	0 (0%)	1 (2.1%)	0 (0%)	
Distended Abdomen						
Absent	0 (0%)	1 (3.0%)	5 (9.1%)	3 (6.3%)	21 (28.8%)	<0.001
Mild	4 (16.0%)	5 (15.2%)	8 (14.5%)	16 (33.3%)	23 (31.5%)	
Severe	21 (84.0%)	27 (81.8%)	42 (76.4%)	29 (60.4%)	29 (39.7%)	
Eye Discharge/Eyelid Inflammation						
Absent	14 (56.0%)	27 (81.8%)	25 (45.5%)	30 (62.5%)	30 (41.1%)	<0.001
Mild	3 (12.0%)	4 (12.1%)	15 (27.3%)	8 (16.7%)	9 (12.3%)	
Severe	8 (32.0%)	2 (6.1%)	15 (27.3%)	10 (20.8%)	34 (46.6%)	
Gait Disorders						
Absent	0 (0%)	0 (0%)	0 (0%)	0 (0%)	0 (0%)	0.984
Mild	17 (68.0%)	24 (72.7%)	40 (72.7%)	34 (70.8%)	50 (68.5%)	
Severe	8 (32.0%)	9 (27.3%)	15 (27.3%)	14 (29.2%)	23 (31.5%)	
Head Piloerection						
Absent	2 (8.3%)	1 (3.1%)	4 (7.5%)	4 (9.3%)	3 (4.3%)	0.127
Mild	8 (33.3%)	9 (28.1%)	10 (18.9%)	17 (39.5%)	12 (17.1%)	
Severe	14 (58.3%)	22 (68.8%)	39 (73.6%)	22 (51.2%)	55 (78.6%)	
Hunched						
Absent	4 (16.0%)	9 (27.3%)	1 (1.8%)	6 (12.5%)	1 (1.4%)	<0.001
Mild	7 (28.0%)	7 (21.2%)	8 (14.5%)	6 (12.5%)	4 (5.5%)	
Severe	14 (56.0%)	17 (51.5%)	46 (83.6%)	36 (75.0%)	68 (93.2%)	
Kyphosis						
Absent	0 (0%)	0 (0%)	0 (0%)	0 (0%)	0 (0%)	0.696
Mild	0 (0%)	0 (0%)	1 (1.8%)	0 (0%)	0 (0%)	

Severe	25 (100%)	33 (100%)	54 (98.2%)	48 (100%)	73 (100%)	
Malocclusions						
Absent	24 (96.0%)	31 (93.9%)	54 (98.2%)	46 (95.8%)	71 (97.3%)	0.588
Mild	1 (4.0%)	0 (0%)	1 (1.8%)	1 (2.1%)	1 (1.4%)	
Severe	0 (0%)	2 (6.1%)	0 (0%)	1 (2.1%)	1 (1.4%)	
Nasal Discharge						
Absent	25 (100%)	33 (100%)	55 (100%)	48 (100%)	73 (100%)	NA
Mild	0 (0%)	0 (0%)	0 (0%)	0 (0%)	0 (0%)	
Severe	0 (0%)	0 (0%)	0 (0%)	0 (0%)	0 (0%)	
Pallor/Cyanosis						
Absent	1 (4.0%)	1 (3.0%)	2 (3.6%)	2 (4.2%)	2 (2.7%)	0.862
Mild	9 (36.0%)	14 (42.4%)	17 (30.9%)	18 (37.5%)	20 (27.4%)	
Severe	15 (60.0%)	18 (54.5%)	36 (65.5%)	28 (58.3%)	51 (69.9%)	
Paralysis						
Absent	24 (96.0%)	32 (97.0%)	53 (96.4%)	48 (100%)	65 (89.0%)	0.205
Mild	1 (4.0%)	1 (3.0%)	1 (1.8%)	0 (0%)	7 (9.6%)	
Severe	0 (0%)	0 (0%)	1 (1.8%)	0 (0%)	1 (1.4%)	
Peri-retro-orbital Swelling						
Absent	23 (92.0%)	32 (97.0%)	43 (78.2%)	43 (89.6%)	58 (79.5%)	0.152
Mild	1 (4.0%)	1 (3.0%)	8 (14.5%)	4 (8.3%)	6 (8.2%)	
Severe	1 (4.0%)	0 (0%)	4 (7.3%)	1 (2.1%)	9 (12.3%)	
Piloerection						
Absent	0 (0%)	0 (0%)	0 (0%)	0 (0%)	0 (0%)	NA
Mild	0 (0%)	0 (0%)	0 (0%)	0 (0%)	0 (0%)	
Severe	25 (100%)	33 (100%)	55 (100%)	48 (100%)	73 (100%)	
Rectal Prolapse						
Absent	25 (100%)	32 (97.0%)	53 (96.4%)	46 (95.8%)	68 (93.2%)	0.991
Mild	0 (0%)	0 (0%)	1 (1.8%)	1 (2.1%)	3 (4.1%)	
Severe	0 (0%)	1 (3.0%)	1 (1.8%)	1 (2.1%)	2 (2.7%)	
Response to Analgesic						
Absent	21 (84.0%)	30 (90.9%)	49 (89.1%)	40 (83.3%)	66 (90.4%)	0.593
Mild	4 (16.0%)	3 (9.1%)	3 (5.5%)	6 (12.5%)	6 (8.2%)	
Severe	0 (0%)	0 (0%)	3 (5.5%)	2 (4.2%)	1 (1.4%)	
Response to External Stimuli						
Absent	25 (100%)	33 (100%)	55 (100%)	48 (100%)	72 (98.6%)	1
Severe	0 (0%)	0 (0%)	0 (0%)	0 (0%)	1 (1.4%)	
Tail Stiffening						
Absent	4 (16.0%)	6 (18.2%)	6 (10.9%)	4 (8.3%)	5 (6.8%)	0.807
Mild	8 (32.0%)	10 (30.3%)	17 (30.9%)	14 (29.2%)	22 (30.1%)	
Severe	13 (52.0%)	17 (51.5%)	32 (58.2%)	30 (62.5%)	46 (63.0%)	
Thoracic Mass						
Absent	12 (50.0%)	19 (59.4%)	19 (35.8%)	23 (53.5%)	27 (38.6%)	0.481
Mild	8 (33.3%)	7 (21.9%)	20 (37.7%)	13 (30.2%)	26 (37.1%)	
Severe	4 (16.7%)	6 (18.8%)	14 (26.4%)	7 (16.3%)	17 (24.3%)	
Tremor						
Absent	0 (0%)	2 (6.1%)	0 (0%)	2 (4.2%)	1 (1.4%)	0.156
Mild	8 (32.0%)	10 (30.3%)	11 (20.0%)	13 (27.1%)	11 (15.1%)	
Severe	17 (68.0%)	21 (63.6%)	44 (80.0%)	33 (68.8%)	61 (83.6%)	
Tumors						
Absent	4 (16.0%)	13 (39.4%)	9 (16.4%)	18 (37.5%)	26 (35.6%)	0.0755
Mild	3 (12.0%)	6 (18.2%)	11 (20.0%)	9 (18.8%)	16 (21.9%)	
Severe	18 (72.0%)	14 (42.4%)	35 (63.6%)	21 (43.8%)	31 (42.5%)	
Urine						
Absent	2 (8.0%)	7 (21.2%)	5 (9.1%)	8 (16.7%)	2 (2.7%)	0.018
Mild	0 (0%)	0 (0%)	0 (0%)	0 (0%)	0 (0%)	
Severe	23 (92.0%)	26 (78.8%)	50 (90.9%)	40 (83.3%)	71 (97.3%)	
Vaginal/Uterine Prolapse						
Absent	25 (100%)	33 (100%)	55 (100%)	48 (100%)	73 (100%)	NA

Mild	0 (0%)	0 (0%)	0 (0%)	0 (0%)	0 (0%)	
Severe	0 (0%)	0 (0%)	0 (0%)	0 (0%)	0 (0%)	
Vestibular Disturbance						
Absent	4 (16.0%)	4 (12.1%)	3 (5.5%)	10 (20.8%)	1 (1.4%)	<0.001
Mild	11 (44.0%)	18 (54.5%)	26 (47.3%)	18 (37.5%)	17 (23.3%)	
Severe	10 (40.0%)	11 (33.3%)	26 (47.3%)	20 (41.7%)	55 (75.3%)	

eTable 3: Cumulative incidence of frailty indicators by sex in aged C57BL/6J mice

	**Males (N=24)**	**Females (N=12)**	**P-value**
Activity			
Absent	9 (37.5%)	12 (100%)	<0.001
Mild	15 (62.5%)	0 (0%)	
Severe	0 (0%)	0 (0%)	
Body Condition			
Absent	1 (4.2%)	0 (0%)	0.82
Mild	10 (41.7%)	6 (50.0%)	
Severe	13 (54.2%)	6 (50.0%)	
Breathing Rate/Depth			
Absent	0 (0%)	1 (8.3%)	0.235
Mild	23 (95.8%)	10 (83.3%)	
Severe	1 (4.2%)	1 (8.3%)	
Changes to Eye Globe			
Absent	23 (95.8%)	10 (83.3%)	0.256
Mild	1 (4.2%)	1 (8.3%)	
Severe	0 (0%)	1 (8.3%)	
Coat Condition			
Absent	0 (0%)	1 (8.3%)	0.1
Mild	1 (4.2%)	2 (16.7%)	
Severe	23 (95.8%)	9 (75.0%)	
Dehydration, Skin Turgor			
Absent	11 (45.8%)	7 (58.3%)	0.727
Mild	0 (0%)	0 (0%)	
Severe	13 (54.2%)	5 (41.7%)	
Dermatitis			
Absent	19 (79.2%)	12 (100%)	0.367
Mild	4 (16.7%)	0 (0%)	
Severe	1 (4.2%)	0 (0%)	
Diarrhea			
Absent	23 (95.8%)	12 (100%)	1
Severe	1 (4.2%)	0 (0%)	
Distended Abdomen			
Absent	1 (4.2%)	1 (8.3%)	0.336
Mild	3 (12.5%)	3 (25.0%)	
Severe	20 (83.3%)	8 (66.7%)	
Eye Discharge/Eyelid Inflammation			
Absent	18 (75.0%)	6 (50.0%)	0.174
Mild	6 (25.0%)	5 (41.7%)	
Severe	0 (0%)	1 (8.3%)	
Gait Disorders			
Absent	0 (0%)	0 (0%)	1
Mild	22 (91.7%)	11 (91.7%)	
Severe	2 (8.3%)	1 (8.3%)	
Head Piloerection			
Absent	1 (4.2%)	4 (33.3%)	0.067
Mild	11 (45.8%)	3 (25.0%)	
Severe	12 (50.0%)	5 (41.7%)	
Hunched			
Absent	0 (0%)	0 (0%)	0.028
Mild	2 (8.3%)	5 (41.7%)	
Severe	22 (91.7%)	7 (58.3%)	
Kyphosis			
Absent	0 (0%)	0 (0%)	0.342
Mild	0 (0%)	1 (8.3%)	

Severe	24 (100%)	11 (91.7%)	
Malocclusions			
Absent	24 (100%)	12 (100%)	NA
Mild	0 (0%)	0 (0%)	
Severe	0 (0%)	0 (0%)	
Nasal Discharge			
Absent	24 (100%)	12 (100%)	NA
Mild	0 (0%)	0 (0%)	
Severe	0 (0%)	0 (0%)	
Pallor/Cyanosis			
Absent	1 (4.2%)	2 (16.7%)	0.499
Mild	15 (62.5%)	6 (50.0%)	
Severe	8 (33.3%)	4 (33.3%)	
Paralysis			
Absent	24 (100%)	12 (100%)	NA
Mild	0 (0%)	0 (0%)	
Severe	0 (0%)	0 (0%)	
Peri-retro-orbital Swelling			
Absent	23 (95.8%)	9 (75.0%)	0.101
Mild	1 (4.2%)	3 (25.0%)	
Severe	0 (0%)	0 (0%)	
Piloerection			
Absent	0 (0%)	0 (0%)	NA
Mild	0 (0%)	0 (0%)	
Severe	24 (100%)	12 (100%)	
Rectal Prolapse			
Absent	24 (100%)	10 (83.3%)	0.109
Mild	0 (0%)	0 (0%)	
Severe	0 (0%)	2 (16.7%)	
Response to Analgesic			
Absent	24 (100%)	12 (100%)	NA
Mild	0 (0%)	0 (0%)	
Severe	0 (0%)	0 (0%)	
Response to External Stimuli			
Absent	24 (100%)	12 (100%)	1
Severe	0 (0%)	0 (0%)	
Tail Stiffening			
Absent	1 (4.2%)	2 (16.7%)	0.147
Mild	10 (41.7%)	2 (16.7%)	
Severe	13 (54.2%)	8 (66.7%)	
Thoracic Mass			
Absent	11 (45.8%)	9 (75.0%)	0.158
Mild	13 (54.2%)	3 (25.0%)	
Severe	0 (0%)	0 (0%)	
Tremor			
Absent	0 (0%)	1 (8.3%)	0.516
Mild	10 (41.7%)	4 (33.3%)	
Severe	14 (58.3%)	7 (58.3%)	
Tumors			
Absent	2 (8.3%)	2 (16.7%)	0.0035
Mild	2 (8.3%)	6 (50.0%)	
Severe	20 (83.3%)	4 (33.3%)	
Urine			
Absent	11 (45.8%)	7 (58.3%)	0.727
Mild	0 (0%)	0 (0%)	
Severe	13 (54.2%)	5 (41.7%)	
Vaginal/Uterine Prolapse			
Absent	24 (100%)	12 (100%)	NA

Mild	0 (0%)	0 (0%)	
Severe	0 (0%)	0 (0%)	
Vestibular Disturbance			
Absent	0 (0%)	2 (16.7%)	0.139
Mild	5 (20.8%)	3 (25.0%)	
Severe	19 (79.2%)	7 (58.3%)	

eTable 4: Correlation coefficients (95% confidence intervals [CI]) and p-values for life expectancy with fragility items by diet in aged J:DO mice

	r AL	p AL	r IF1	p IF1	r CR20	p CR20	r IF2	p IF2	r CR40	p CR40
Body Condition	-0.40	<0.001	-0.34	<0.001	-0.33	<0.001	-0.36	<0.001	-0.26	<0.001
Body Weight	0.33	<0.001	0.35	<0.001	0.14	<0.001	0.42	<0.001	0.28	<0.001
Tail Stiffening	-0.30	<0.001	-0.24	<0.001	-0.10	0.001	-0.25	<0.001	-0.28	<0.001
Head Piloerection	-0.29	<0.001	-0.21	<0.001	-0.24	<0.001	-0.35	<0.001	-0.31	<0.001
Gait Disorders	-0.29	<0.001	-0.24	<0.001	-0.10	0.001	-0.08	0.042	-0.12	<0.001
Tremor	-0.28	<0.001	-0.16	0.001	-0.26	<0.001	-0.15	<0.001	-0.27	<0.001
Temperature	0.27	<0.001	0.26	<0.001	0.24	<0.001	0.30	<0.001	0.27	<0.001
FgI Score	-0.26	<0.001	-0.26	<0.001	-0.37	<0.001	-0.33	<0.001	-0.37	<0.001
Eye Discharge/Eyelid Inf.	0.24	<0.001	0.17	<0.001	-0.18	<0.001	-0.05	0.23	-0.14	<0.001
Tumors	-0.23	<0.001	-0.12	0.014	-0.26	<0.001	-0.24	<0.001	-0.14	<0.001
Age in weeks	-0.21	<0.001	-0.35	<0.001	-0.44	<0.001	-0.37	<0.001	-0.43	<0.001
Coat Condition	-0.20	<0.001	-0.17	0.001	-0.29	<0.001	-0.18	<0.001	-0.23	<0.001
Breathing Rate/Depth	-0.19	<0.001	-0.25	<0.001	-0.29	<0.001	-0.23	<0.001	-0.19	<0.001
N Deficits	-0.19	<0.001	-0.21	<0.001	-0.34	<0.001	-0.29	<0.001	-0.35	<0.001
Changes to Eye Globe	0.18	0.001	0.28	<0.001	-0.04	0.14	-0.11	0.005	-0.12	<0.001
Kyphosis	-0.16	0.003	-0.15	0.002	-0.08	0.004	-0.09	0.02	-0.10	<0.001
Hunched	-0.16	0.003	-0.35	<0.001	-0.18	<0.001	-0.22	<0.001	-0.23	<0.001
Thoracic Mass	-0.16	0.003	-0.10	0.036	-0.14	<0.001	-0.13	0.001	-0.05	0.012
Dermatitis	-0.15	0.004	-0.12	0.013	-0.06	0.029	-0.18	<0.001	-0.08	<0.001
Dehydration, Skin Turgor	-0.14	0.012	-0.03	0.53	-0.12	<0.001	-0.14	<0.001	-0.14	<0.001
Pallor/Cyanosis	-0.13	0.016	-0.10	0.041	-0.11	<0.001	-0.21	<0.001	-0.21	<0.001
Distended Abdomen	0.10	0.053	0.03	0.6	0.07	0.012	0.18	<0.001	0.03	0.19
Malocclusions	-0.06	0.25	-0.04	0.47	-0.03	0.34	-0.01	0.7	0.01	0.78
Diarrhea	-0.04	0.44	-0.05	0.31			-0.04	0.25		
Response to Analgesic	-0.02	0.67	-0.07	0.17	-0.06	0.047	0.13	0.001	-0.06	0.003
Peri-retro-orbital Swelling	-0.02	0.68	-0.06	0.21	-0.08	0.006	-0.12	0.002	-0.05	0.015
Piloerection	-0.01	0.79	-0.05	0.33	-0.08	0.008	-0.14	<0.001	-0.04	0.037
Activity	0.01	0.77	0.01	0.9	0.00	0.93	-0.04	0.28	-0.03	0.12
Paralysis	-0.01	0.86	-0.03	0.5	-0.06	0.029			-0.11	<0.001
Vestibular Disturbance	-0.01	0.89	0.00	0.94	-0.05	0.09	-0.01	0.75	-0.07	<0.001
Urine	0.01	0.92	-0.01	0.77	-0.10	<0.001	-0.11	0.004	-0.12	<0.001
Nasal Discharge										
Rectal Prolapse			-0.05	0.31	-0.05	0.11	-0.09	0.019	-0.07	<0.001
Vaginal/Uterine Prolapse										
Response to External Stim.									-0.03	0.18

Items ordered by descending absolute correlation coefficient in AL cohort; r=Pearson correlation, Eyelid Inf. = Eyelid Inflammation, Stim. = Stimuli.

eTable 5: Correlation coefficients (95% confidence intervals [CI]) and p-values for life expectancy with fragility items by sex in aged C57BL/6J mice

	r Females	p Females	r Males	p Males
Age in weeks	-0.45	$<$0.001	-0.47	$<$0.001
FgI Score	-0.44	$<$0.001	-0.42	$<$0.001
Body Condition	-0.44	$<$0.001	-0.37	$<$0.001
Pallor/Cyanosis	-0.40	$<$0.001	-0.23	$<$0.001
Head Piloerection	-0.39	$<$0.001	-0.39	$<$0.001
N Deficits	-0.34	$<$0.001	-0.32	$<$0.001
Coat Condition	-0.34	$<$0.001	-0.14	$<$0.001
Body Weight	0.33	$<$0.001	0.09	0.02
Hunched	-0.33	$<$0.001	-0.29	$<$0.001
Tremor	-0.33	$<$0.001	-0.30	$<$0.001
Tumors	-0.26	$<$0.001	-0.36	$<$0.001
Eye Discharge/Eyelid Inflammation	-0.25	$<$0.001	-0.12	$<$0.001
Temperature	0.24	$<$0.001	0.28	$<$0.001
Rectal Prolapse	-0.22	$<$0.001		
Urine	-0.21	$<$0.001	-0.09	0.03
Dehydration, Skin Turgor	-0.21	$<$0.001	-0.13	$<$0.001
Tail Stiffening	-0.18	0.01	-0.17	$<$0.001
Breathing Rate/Depth	-0.17	0.01	-0.19	$<$0.001
Distended Abdomen	0.17	0.01	-0.21	$<$0.001
Changes to Eye Globe	-0.16	0.01	0.03	0.37
Peri-retro-orbital Swelling	-0.15	0.03	0.02	0.54
Kyphosis	-0.14	0.04	-0.10	0.01
Vestibular Disturbance	0.13	0.04	0.06	0.13
Gait Disorders	-0.07	0.32	-0.18	$<$0.001
Thoracic Mass	0.05	0.46	-0.05	0.21
Piloerection	-0.02	0.76	0.06	0.1
Dermatitis			-0.13	$<$0.001
Response to Analgesic				
Nasal Discharge				
Vaginal/Uterine Prolapse				
Diarrhea			0.03	0.48
Activity			0.04	0.31
Response to External Stimuli				
Paralysis				
Malocclusions				

Items ordered by descending absolute correlation coefficient in Female cohort; r=Pearson correlation.

eTable 6: Model performance for TxW predictive endpoint criteria across two genetic backgrounds, both sexes, and five experimental groups.

Strain	Sex	Diet	Predicted	Observed	Sensitivity	Specificity	NPV	PPV
C57BL/6J	M	AL	85	109	0.28	0.84	0.79	0.36
C57BL/6J	F	AL	82	53	0.93	0.48	0.89	0.60
J:DO	F	IF1	85	116	0.53	0.61	0.40	0.73
J:DO	F	CR20	280	290	0.45	0.67	0.66	0.46
J:DO	F	IF2	130	174	0.53	0.74	0.56	0.71
J:DO	F	CR40	391	434	0.26	0.80	0.77	0.29
J:DO	F	AL	55	70	0.27	0.46	0.38	0.34

Note: Abbreviations: NPV = negative predicted value, PPV = positive predicted value, TxW = the product of temperature and body weight. PPV is the probability that, following a positive prediction, the sample is truly positive. NPV is the probability that, following a negative prediction, the sample is truly negative. TxW performance required 13+ weeks of data.
